# Managers’ experiences in leading healthcare workers in hospital departments during a pandemic: a qualitative study

**DOI:** 10.1186/s12913-025-12759-w

**Published:** 2025-05-13

**Authors:** Linda Ahlstrom, Nanna Gillberg, Ewa Wikström, Helle Wijk, Ingibjörg H. Jonsdottir, Alessio Degl’Innocenti, Sara L. Fallman

**Affiliations:** 1 Institute of Health and Care Sciences, Sahlgrenska Academy, University of Gothenburg, Gothenburg, Sweden; 2 Department of Orthopaedics, Sahlgrenska University Hospital, Region Västra Götaland, Gothenburg, Sweden; 3 The School of Business, Economics and Law, Department of Business Administration, University of Gothenburg, Gothenburg, Sweden; 4 Department of Quality Strategies, Sahlgrenska University Hospital, Region Västra Götaland, Gothenburg, Sweden; 5 Department of Architecture and Civil Engineering, Chalmers University of Technology, Gothenburg, Sweden; 6 Institute of Stress Medicine, Region Västra Götaland, Gothenburg, Sweden; 7 School of Public Health and Community Medicine, Institute of Medicine, Sahlgrenska Academy, University of Gothenburg, Gothenburg, Sweden; 8 Region Västra Götaland, Regionhälsan, Gothenburg, Sweden; 9 Centre for Ethics, Law, and Mental Health (CELAM), Department of Psychiatry and Neurochemistry, Institute of Neuroscience and Physiology, Sahlgrenska Academy, University of Gothenburg, Gothenburg, Sweden; 10 Department of Work Life and Social Welfare, University of Borås, Borås, Sweden

**Keywords:** Health services research, COVID- 19, Crises management, Leadership, Qualitative research

## Abstract

**Background:**

The COVID-19 pandemic exposed critical weaknesses within the healthcare sector in many countries, and intensifying pressure on managers and healthcare workers was immediate. Managers were faced with increased pressure during the pandemic related to difficult prioritizations and severely disrupted daily operations. Therefore, a deeper understanding of managers’ similar and different experiences during pandemics is necessary. Our aim of this study was to explore the experiences of managers’ in leading HCWs at two major Swedish hospitals during the COVID-19 pandemic. Further, to describe their applied managerial approaches and strategies, and comparing the experiences of managers overseeing units caring for COVID-19 patients with those managing units not involved in COVID-19 patient care, to identify both differences and similarities in their crisis management practices.

**Method:**

A qualitative research design involving thematic analysis was applied during the COVID-19 pandemic. Data used in this study were from the open-ended questions in a web-based survey; 376 managers were included.

**Results:**

Managers´ experiences in leading HCWs resulted in four themes: (1) centralised crisis management, (2) managing daily operative work, (3) dynamics of managerial support, and (4) new insights into learning and development, accompanied by 13 subthemes. Their experiences in leading HCWs caring for COVID-19 patients or not varied, and managers need to recognize these variations in strategies to provide effective and crucial support to HCWs during challenging times. Managers also appreciated certain hospital operations during the pandemic, while identifying areas for improvement within the organisation.

**Conclusions:**

Acknowledging diverse needs among managers is important, especially given the diverse conditions in departments caring for COVID-19 patients versus others. Managers play a critical role in centralised crisis management, ensuring daily operative work, providing managerial support to healthcare workers, and fostering a culture of continuous learning and development. These insights highlight the importance of effective management and the dynamics of managerial support during pandemic crises, which directly impact patient safety. These results contribute to the ongoing improvement of healthcare organisations in addressing unforeseen challenges, such as pandemics and broader existential crises, by emphasizing the managerial perspective on crisis response.

**Supplementary Information:**

The online version contains supplementary material available at 10.1186/s12913-025-12759-w.

## Background

The COVID- 19 pandemic revealed critical weaknesses in healthcare systems, initially catching healthcare organisations off guard [1, 2], with difficulties in balancing the need of COVID- 19 patients as well as other patients [3]. Managers and healthcare workers faced extraordinary challenges, during the crises, intensifying pressure on managers and impacting entire hospital organizations [4]. Studies have shown that hospital managers encountered numerous challenges during the pandemic that they had to manage, including difficulties with communication [2], fears of infection [5], worries [6] and anxiety among employees [7]. Furthermore, the focus on acute COVID- 19 care led to temporary suspension of ordinary patient care, including cancer screening and elective surgery, causing concern among managers [8]. Data presented by the Swedish National Board of Health and Welfare [9], confirmed a decrease in surgeries, cases of inpatient somatic care, and outpatient surgical procedures during the pandemic compared to pre-pandemic levels. This reduction poses significant challenges for healthcare managers, who had to navigate resource allocation, staff shortages [10], and patient prioritization in an already strained situation [11].

Within a hospital organisation, a crisis can be conceptualized as a problematic and disruptive situation that surpasses the capacity of routine managerial strategies to resolve effectively [12]. Crisis may severely impact the organisation’s normal functioning and stability as well as require immediate strategic interventions from mangers to mitigate the crisis’s potential negative effects on their HCWs and patient safety [13, 14]. To clarify and guide these critical responses, crisis management theory includes models of processes that organisations, and crucially the managers, can employ when confronting a crises or disruptive event [15]. At such times, managers still have to prioritise daily hospital operations, manoeuvre across organisational levels, meet set targets, and engage in continuous learning and development, which they can ideally achieve by following the system theory approach [16]. The system theory approach recognises the interconnectedness of HCWs, resources, organisations, and policies that constitute a given healthcare system. Applying the approach may be particularly suitable during a crisis, as it aids healthcare organizations and managers in making informed decisions while considering their impact on the entire healthcare system. By gathering information from different sources, managers can identify areas for improvement and make informed decisions and implement necessary changes to optimise their operations during crises [17]. This approach supports managers in moving forward and cultivating sustainable working conditions and high-quality care [18].

In that context, sustainable working conditions could be defined as conditions when the managers actively support the development of resources for HCWs [19, 20]. The manager’s role is crucial to enhance their work situation and, consequently, their ability to remain at work [17]. For example, as the COVID- 19 pandemic continued to significantly impact healthcare organisations and required them to adapt rapidly to constantly changing demands and conditions [21] resilience became crucial for sustainable working conditions for HCWs. On that count, organisational resilience is an organisation’s ability to adapt, learn, and grow in the face of uncertainty, insecurity, and rapid transformation, which is of significant importance in healthcare organisations [22, 23]. At base, a resilient healthcare organisation effectively aids HCWs in anticipating, adjusting to, coping with, and recovering from adverse circumstances, thereby enabling them to deliver high-quality healthcare. During crises, managers, whether in healthcare or otherwise, are expected to be strategic and flexible in creating sustainable working conditions, while also grounding their management on holistic approaches and encompassing pedagogical principles focused on individual employees [21]. In hospitals, managers are expected to utilise their capability to adapt and foster healthy organisations [16]. Furthermore, a quantitative study conducted as part of the same research project as this one demonstrates the varied impacts of the pandemic on hospital departments in varied ways, underscoring highlighting its complex nature. Crucial concerns during periods of heightened workload, such as the pandemic, involve notably insufficient managerial support and inadequate recuperation time while on duty [24].

Existing research underscores the importance of managerial roles, systems theory, and resilience in healthcare crisis management. However, a detailed analysis of the specific managerial strategies and approaches enacted during the COVID- 19 pandemic is lacking. This research addresses this deficiency by investigating these strategies and approaches, aiming to identify the specific managerial experiences and their impact, providing practical insights for future crisis management in healthcare.

## Methods

### Aim

Given the necessity for additional knowledge of the challenges faced by managers leading HCWs during crises, such as the COVID- 19 pandemic, this study aims to explore the experiences of managers in hospital departments during such crises. It specifically focuses on identifying their applied managerial approaches and strategies, and comparing the experiences of managers overseeing units caring for COVID- 19 patients with those managing units not involved in COVID- 19 patient care, to identify both differences and similarities in their crisis management practices.

### Design and setting

The study followed a qualitative research design involving thematic analysis [25] conducted at one university and one county hospital. The study was conducted at two hospitals in Sweden: a university hospital and a county hospital. The university hospital, employing approximately 17,000 individuals, is among the largest in Sweden, while the county hospital, with a workforce of around 4000 thousand employees, serving the healthcare needs of a local population equivalent to that of a small city. Both hospitals played a vital role in delivering frontline care to patients with COVID- 19 during the pandemic. This study was part of a larger research project investigating the work environments and health of HCWs during the first wave of COVID- 19 in large hospitals in Sweden. Comprehensive detailed description of the project, participants, response rates and the questionnaire, has been previously published in Jonsdottir et al. [24]. The project’s diverse research group has comprised individuals from various professions with different levels of work experience in Sweden, both at universities, and within healthcare.

To improve our study’s transparency, we followed the Standards for Reporting on Qualitative Research (SRQR) described by O’Brien et al. [26]. The study was also conducted in compliance with the Declaration of Helsinki [27] and approved by the Swedish Ethical Review Authority (Ref. No. 2020–04771), and participants provided their informed consent by responding to a specific question in the web-based survey. Data were collected using a web-based survey sent via email and provided information about the study and additional guidelines regarding the questionnaire.

### Participants and data collection

The web-based survey was administered in collaboration with the hospital’s Human Resources department to all hospital employees including managers and was designed to be completed within 10–20 min. Participants were asked to reflect on how they perceived the situation during the intensive period of the pandemic in spring 2020 when answering questions about their work conditions. The first section of the survey consisted of demographic items and work conditions, addressing work demands, support, recovery, and engagement. One part of the survey addresses the participants mental and physical health and whether they have received any support from the employer during the pandemic. Additional items about work placement during the pandemic, worries about getting infected, and access to personal protective equipment were also included. The final section of the survey, used for this qualitative study, consisted of three open-ended questions, starting with a screening question: Are you a manager? (Yes/No). Managers were then asked to share their experiences about (1) important, (2) insufficient, (3) positive and negative experiences concerning organisational prerequisites that were particularly important or lacking during the onset of the COVID- 19 pandemic in the spring of 2020. These open-ended questions are presented in Supplementary file 1, as also including the inclusion criteria question about working as manager, caring for COVID- 19 patients or not, and descriptive data questions.

Of the web-based survey, 454 of the respondents were managers, 376 (86%) provided at least one open-ended response (free-text answer), which constituted the data for this study. Descriptive data are presented in Table 1. Managers were divided into two groups: managers leading HCWs in a department caring for patients with COVID- 19, called “COVID- 19 managers” (*n* = 74, 20%), and managers leading HCWs who were not caring for such patients (*n* = 302, 80%), called “non-COVID- 19 managers”. About three out of four (77%) managers were females, and most of them between 40 and 59 years (73%).

**Table 1:** Descriptive data of the managers included in the qualitative study

Variables	University Hospital	County Hospital	Total
	Sample/ Percentage^*^ N (%)	Sample/ Percentage^*^ N (%)	Sample/ Percentage^**^ N (%)
Replied to web-based survey			
Managers	390 (86)	64 (14)	454 (6)
Included in this study			
Managers with free-text answers	318 (85)	58 (15)	376 (83)
Manager groups; (*departments caring for patients with COVID- 19 and those not)*			
COVID- 19 managers	63 (85)	11 (15)	74 (20)
non-COVID- 19 managers	255 (84)	47 (16)	302 (80)
Distribution by gender			
Female	250 (86)	41 (14)	291 (77)
Male	65 (80)	16 (20)	81 (23)
Prefer not to say/missing data	3 (75)	1 (25)	4 (0,1)
Distribution by age			
≥ 60	52 (90)	6 (10)	58 (15)
50–59	135 (85)	23 (15)	158 (42)
40–49	95 (81)	23 (19)	118 (31)
30–39	34 (85)	6 (15)	40 (11)
≤ 29	0	0	0
Missing data	2 (< 1)	0	2 (< 1)

^*^Percentage calculated of numbers between hospitals

^**^Percentage calculated out of total numbers

### Data analysis

Following Braun and Clarke [25] method, the initial phase of thematic analysis involved an in-depth review of the data by two authors (LA and SLF). The data were actively reviewed individually and collaboratively, in an iterative process, to gain a comprehensive understanding of their importance. During the process, notes were taken to capture initial thoughts, ideas, and impressions. Afterward, initial codes were generated, and the data were coded by identifying relevant features and patterns. Next, a thematic search was performed to identify similarities for the extraction of data and patterns across the initial codes, and the codes were grouped into subthemes and grouped into themes (Table 2). Ensuring the accuracy and alignment of the data was a crucial next step; although the method employed has been called a linear one, we performed an iterative, reflective process that evolved and required movement back and forth between all these steps as described by Nowell et al. [28]. To reach consensus, further deliberations occurred within the broader project’s research group with two other authors (i.e. EW and NG). Afterward, themes were reviewed and redefined into new themes. The final phase of analysing the data engaged all authors in discussions regarding subthemes and themes to ensure synchronisation with the data. All data in the dataset were read and reviewed, as recommended by Braun and Clarke [25], to affirm the alignment and the essence of the meaning of each theme.

**Table 2:** Example of data extraction, codes, subtheme, and theme

Data extraction	Codes	Subtheme	Theme
We were given space to improvise and do what we were good at.	Freedom to make decisions in daily work.	Empowered decision-making	Managing daily operative work

For a trustworthy thematic analysis, we followed and implemented rigorous, systematic processes that can be verified for quality, credibility, and transferability. Key considerations for ensuring trustworthiness were ensuring the transparency and documentation of the analysis. For an external validation to enhance the rigor of and confidence in our findings [26, 28], we sought a peer review of our analysis within the broader project’s research group. Last, to reduce errors in interpretation, we reflected on our personal biases and assumptions and recognised the crucial impact of researcher reflexivity, meaning a researcher’s critical self-reflection on their personal background, preferences, and preconceptions and how those influences may impact analysis [28].

## Results

The focus of the study was to explore the experiences of managers at two major Swedish hospitals during the COVID- 19 pandemic. Additionally, the study aimed to identify potential differences between managers overseeing departments caring for patients infected with COVID- 19, and managers not caring for patients infected with COVID- 19. The analysis of the data resulted in four themes: (1) Centralized crisis management (2) managing daily operative work, (3) dynamics of managerial support, and (4) new insights into learning and development, accompanied by 13 subthemes. An overview of the themes and subthemes is presented in Table 3.

**Table 3:** Managers’ experiences in leading healthcare workers during the COVID- 19 pandemic: themes and subthemes

Themes	Subthemes
Centralized crisis management	Top-down direction of orders Rapid decisions and diverse directives Information overload
Managing daily operative work	Empowered decision making Challenges in resource allocation and staffing Crisis-driven collaboration and teamwork
Dynamics of managerial support	Supporting healthcare workers (HCWs) wellbeing Administrative burden Limited human resources operational support Valued digital support Need for support, recognition, and appreciation
New insights into learning and development	Crisis-driven innovation Need for strategic crisis readiness

### Centralized crisis management

The first theme consists of three subthemes: top-down directions of orders, rapid decisions and diverse directives, and information overload. Centralized crises management involved a shift towards a more top-down management, allowing less room for interaction with top management. Additionally, rapid decisions from top management were crucial due to the time sensitivity in saving patients with COVID- 19, although sometimes accompanied by diverse directives. As new information emerged, top management shared an overload of information, sometimes through multiple email communications per day.

#### Top-down directions of orders

Managers in both groups highlighted that the hospitals’ top management had become more authoritarian during the COVID- 19 pandemic. They experienced a demand for action and requirements for feedback on results provided by top management, which were followed up rapidly, often daily.

*“The overall structure*,* decision-making*,* and information from top management downwards worked well. It felt nice to know that the organization could step up*,* make decisions*,* and set frameworks but also that great trust existed in the work being done.”* (Non-COVID- 19 manager)

COVID- 19 managers emphasized the importance of top-down management, which they characterized as clear and concise, and they could act promptly on orders. As the first wave of COVID- 19 intensified, the hospitals’ top management shifted focus from finances to saving lives, which made the managers’ work more manageable, at least according to the COVID- 19 managers:

*“Clear governance from top management and good support from the immediate manager have been important prerequisites in my work as a manager during the pandemic.”* (COVID- 19 manager)

Meanwhile, non-COVID- 19 managers often perceived that directives and objectives did not consistently align with the specific requirements and demands of their respective departments. Some non-COVID- 19 managers expressed a desire for more comprehensive guidelines from the management to assist them in achieving their goals.

#### Rapid decisions and diverse directives

Managers in both groups noted a strong inclination towards rapid decisions and diverse directives by top management. The acceleration in decisions was perceived as something completely different from the typically slow, ponderous decision process followed before the pandemic. The situation led to frequent adjustments in routines because decisions often lacked thorough consideration. Sudden, daily changes in guidelines posed challenges and made it difficult for managers to stay well-informed. Such rapid decisions from management prompted continuous changes, a trial-and-error approach, and occasional managerial fatigue.

*“What was positive during the pandemic was the rapid decisions and a way of working in which we tested*,* and if necessary*,* changed what was not working.”* (non-COVID- 19 manager)

The clarity in directives was mentioned by both groups of managers as an important organizational prerequisite. Managers wanted top management to be even more direct about who was responsible for different roles and more concise about who was responsible for decision-making.

#### Information overload

Both groups stated the importance of information to be clear, uniform, and targeted. Managers had to skim through the overload of information to forward relevant information to their HCWs. They described the challenge of maintaining accurate and up to date information, particularly regarding COVID- 19 guidelines for patient care, the accuracy of which they felt pressured to ensure. They also reported spending a significant amount of time screening for targeted information and crucial knowledge for their HCWs, even as the entire organization was facing considerable time pressure. The COVID- 19 managers requested and desired for more hands-on and pinpointed information relevant to their departments.

*“As a manager*,* you print information*,* and distribute it through notes on the wall*,* orally*,* and in pamphlets. You had to keep calm and carry on.”* (COVID- 19 manager)

The non-COVID- 19 managers, on the other hand, had a great need for unfiltered and broader information about the pandemic to gain the full picture.

### Managing daily operative work

The second theme consists of three subthemes: empowered decision making, challenges in resource allocation and staffing, and crisis-driven collaboration and teamwork. On a daily basis, the managers experienced increased empowered decision making, which involved restructuring their daily operations, and they further struggled with allocating resources and staffing departments. The managers expressed being impressed by the HCWs’ commitment and flexibility in completing work tasks daily during the pandemic, which they said impacted their involvement and motivation to work collaborative as a team.

#### Empowered decision making

Regarding empowered decision making, although the managers’ experiences were diverse, it emerged in both groups that the managers had been given the power to make their own decisions within their units. They had the mandate to design and influence the work of their units in ways that were best for their HCWs and the hospital organization. This empowerment made them feel the trust of top management in their license to make decisions:

*“We were given space to improvise and do what we’re good at.”* (COVID- 19 manager)

COVID- 19 managers felt that they were the centre of attention and given room to manoeuvre. They also emphasized the impossibility of performing daily operations during the pandemic without their increased autonomy regarding decision-making.

*“My overall impression was that*,* as a professional and manager*,* I had the freedom and room to manoeuvre that I’d never experienced before in my professional life.”* (COVID- 19 manager)

Non-COVID- 19 managers, by comparison, highlighted the potential downside of excessive autonomy regarding empowered decision-making and expressed concerns about the associated rise in ambiguity regarding their responsibilities.

#### Challenges in resource allocation and staffing

Managers from both groups indicated a desire for more concise information on the mobilization between departments of staffing resources for HCWs. COVID- 19 managers expressed a need for increased predictability and a more holistic approach towards the allocation of HCWs resources. They were concerned about their HCWs, as they frequently had to alter their schedules and postpone time off on short notice.

*“One lesson to draw … is that staffing could’ve been better coordinated and made based on the hospitals’ total needs. In certain periods*,* there were too many staff for COVID- 19-related care and desperately too few staff for regular operations.”* (COVID- 19 manager)

Non-COVID- 19 managers noted the challenge of motivating HCWs to allocate to COVID- 19 wards. They speculated that the difficulty may have arisen from the initial optional nature of the request, which later shifted towards a more coercive approach. As the pandemic progressed, the managers had the feeling that some HCWs experienced the wards with patients infected by COVID- 19 as so well-staffed that they had become crowded.

*“It was difficult to motivate staff to walk away from their units where there were multiple gaps to go to a unit where … there was a surplus of staff*,* and they weren’t needed.”* (Non-COVID- 19 manager)

#### Crisis-driven collaboration and teamwork

The managers in both groups experienced a united front and collaboration at all organizational levels and teamwork between professions. Everyone had a great desire to support each other. The boundaries between the units that they had formerly experienced could be dissolved and established teamwork were more easily crossed. The attitude among the managers was that the HCWs had performed wonderfully in shifting workplaces and collaborating with new teams.

*“It was a great experience to be able to help when Sweden was put to the test. It was positive that we managed the situation and that the patients received good care and survived.” (COVID- 19 manager)*

Non-COVID- 19 managers reported that they worked hard to promote a feeling of teamwork even as HCWs continually joined and left their departments, while HCWs had to work at COVID- 19 departments. Their experience was that they did not want to be forgotten but would like to be more included in the community and acknowledged as being important as well, fostering a sense of collaboration across departments.

*“The solidarity and courage of individual employees were impressive! There was a willingness to cooperate across unit boundaries*,* and stories now testify to positive experiences that the staff wants to build upon.” (Non-COVID- 19 manager)*

The non-COVID- 19 managers felt that it was essential that they and their HCWs felt included in the “war zone” and were not forgotten.

### Dynamics of managerial support

The third theme, dynamics of managerial support, unveiled a landscape of five subthemes: supporting HCWs wellbeing, administrative burden, limited human resources operational support, valued digital support, and managers own need for support, recognition and appreciation. The overarching findings reveal that during the dynamics of managing crises, managers navigated through a jungle of support functions, recognizing that these vital resources are essential for effective managerial work. Importantly, the findings underscore managers’ own need for support and recognition is crucial, as their wellbeing directly influences their capacity to lead and support HCWs wellbeing effectively.

#### Supporting healthcare workers wellbeing

Both groups of managers acknowledged that a high workload among employees had been challenging and that the HCWs thus needed considerable support. Differences between the two groups were that COVID- 19 managers highlighted the successful provision of PPE as fostering a sense of safety for their HCWs. By contrast, non-COVID- 19 managers faced employees’ concerns regarding inadequate PPE and fear of contracting COVID- 19. Although managers in both groups reported spending significant time listening to and emotionally supporting their HCWs, non-COVID- 19 managers appeared to place even greater emphasis on supporting employee’s fear of being infected.

*“I’ve seen that employees can get so incredibly scared*,* which I didn’t expect*,* and this can be paralyzing for some. I will take this with me in the future as an important experience. You must be sensitive to that fear in similar situations in the future.” (Non-COVID- 19 manager)*

#### Administrative burden

Managers in both groups expressed a lack of administrative support in their daily work. The managers reported having to spend an unreasonable amount of time on contact tracing, booking appointments for COVID- 19 tests, and scheduling staff, among other tasks. These were tasks that they believed could have been handled more efficiently by the administrative department.

*“I needed a secretary. I was drowning in administrative tasks; the amount of small administrative tasks was exorbitant and*,* on top of that*,* required the use of managerial skills.”* (Non-COVID- 19 manager)

The COVID- 19 managers reported appreciating support from specific individuals and often referenced those individuals by name.

#### Limited human resources operational support

The managers in both groups raised concerns about the limited HR operational support, which they characterized as being somewhat disconnected from their specific daily needs. They desired more direct involvement from HR in their daily operative work, to engage in HCWs wellbeing, wishing for them to act. Overall, there was a consensus among managers that HR should take a more proactive role in determining which HCWs should be relocated to different wards, and they cited the need for competence, legislative knowledge, and an overall understanding of the organization.

*“HR should know better and be able to switch to supporting HCWs more closely in clinical work. Here [at my hospital]*,* managers and leaders got way too much placed in their lap when they had to deal with HCWs’ anxiety and their anxiety and mobilize resources*,* which took up all their time.”* (COVID- 19 manager)

#### Valued digital support

Both groups of managers expressed that the information technology department had made additional efforts to provide valued digital support and implement innovative solutions during challenging circumstances. This digital support was highly valued and appreciated by both groups. Initiatives and innovations that would normally take a long time to implement in the organisation had been implemented rapidly and received well during the pandemic. The fact that the pandemic forced a transition to virtual meetings and the opportunity for such meetings was seen as a positive change:

*“The transition to digitalization was positive. Things that had been dragging on for years could suddenly be done in a couple of weeks.”* (COVID- 19 manager)

The non-COVID- 19 managers, to a greater extent than the COVID- 19 managers, mentioned the importance of digitalization during the pandemic and described opportunities to work from home as a positive element of work:

*“Finally*,* we were able to introduce more virtual healthcare meetings and other solutions for e-health*.” (Non-COVID- 19 manager)

#### Need for support, recognition, and appreciation

The perceived need for support, recognition and appreciation described by the managers seems to have varied between the two groups. COVID- 19 managers emphasized the significance of support and recognition from their immediate superiors and other managers, especially given the extended and demanding work hours. Managers highlighted the value of mutual support and recognition among themselves, which they found both beneficial and validating. However, managers from both groups felt an expectation to exert additional effort without receiving the appreciation or compensation they believed they deserved. Most emphasized a strong desire for acknowledgment and appreciation of their efforts:

*“There was no one there for us.” (COVID- 19 manager)*

### New insights into learning and development

The fourth theme, new insights into learning and development, consists of two subthemes: embracing new work models and the importance of crises preparedness. Managers had the impression they encouraged and promoted learning and development to assist HCWs in promptly acquire new skills and knowledge. Additionally, they highlighted the organization’s inadequate preparedness for crises.

#### Crisis-driven innovation

Managers in both groups reported encouraging HCWs to develop new ways of working to empower their growth and develop their knowledge, skills, and capabilities to optimise their performance. They gave no strict guidelines about what the HCWs could do but instead told them to be innovative. During the pandemic, there was a more tolerant climate for trial and error with new methods, although of course with patient safety as an overarching goal. HCWs were effective under such circumstances despite ambiguous information and the frequent absence of supporting documents. Working groups that managed innovative crisis-driven work seemed to have strengthened their groups’ morale and consolidated sense of professionalism into a clear, common whole, embracing the challenge and opportunities presented by the crises:

*“Some things in the organisation that were previously difficult to implement were resolved quickly and efficiently.”* (Non-COVID- 19 manager)

There was strong alignment across all levels, with both HCWs and the organisation displaying enthusiasm to address and solve problems and a sense of coping. It was observed that this period had been highly instructive, and the experience was recognised as having significant value for shaping their future roles as managers.

#### Need for strategic crisis readiness

A mutual reflection of all managers was their surprise at the lack of strategic plans within the organisation in place for handling events such as pandemics. The managers mentioned the need to be prepared and have readiness for upcoming crises and to have structures already in place. Without those measures, the hospital organisations were breeding grounds for confusion, marked by unclear decisions and mixed messaging that made managers seem unprofessional. However, this situation improved over the course of the pandemic, and managers felt more confident and prepared about it over time:

*“We need to learn from this pandemic crisis by having the right skills overall and people who can handle disasters and by applying the right skills in the right place in the event of similar events*,* for relief before people hit the wall.”* (COVID- 19 manager)

## Discussion

The findings reveal both similarities and differences in how managers of HCWs caring for COVID- 19 patients experienced their work situation during the pandemic, compared to managers responsible for HCWs not involved in such care. Managers faced centralised crisis management challenges, including a shift towards a top-down direction of orders, rapid decisions, the need to adapt to frequent changes in diverse directives. Recognizing the challenge of information overload, both COVID- 19 and non-COVID- 19 managers highlighted the need for a clear flow of information, while also acknowledging the differing levels of detail required by different departments. Managers had to restructure their operative daily work to accommodate for upcoming events, and to adopt to empowered decision making. COVID- 19 managers, in particular expressed appreciation for their HCWs’ commitment and flexibility. During the start of the COVID- 19 pandemic, managers observed strong teamwork and togetherness among their HCWs, underscoring the critical importance of collaboration during such extraordinary circumstances. Managers cultivated the sense of unity between themselves and HCWs, which played a pivotal role not only in maintaining the quality of patient healthcare and ensuring patient safety but also in nurturing support among employees, as also found by Vázquez-Calatayud et al. [2].

Managers had challenges in allocating resources for HCWs, however; COVID- 19 managers sought increased predictability, while non-COVID- 19 managers struggled to motivate HCWs to relocate to COVID- 19 wards and emphasised the need for clearer information on the mobilisation of resources, underscoring their wish for more robust HR operational support. Collaboration and teamwork thrived across healthcare units during the crisis and thus broke down pre-existing boundaries. Managers also highlighted the critical and dynamic role of managerial support, encompassing the challenges of the administrative burden, the limitations of HR operational support, and the benefits of valued digital support, which, while helpful, had room for improvement. Furthermore, the results revealed that managers encouraged learning and development among HCWs to adapt to new and innovative crisis work models.

The overall finding emphasises the need for robust strategic crisis readiness, strategic planning, and comprehensive support system to enhance organisational resilience in future crises [22, 23]. In this context, resilience encompasses an organization’s ability to manage daily operative work and adapt to disruptive events, through crises-driven innovation, sustain operational effectiveness and even learn and develop through the crisis. A key aspect of building this resilience is ensuring that top management takes an active role in crisis readiness. This involves not only increasing organizational awareness of potential crises but also providing tailored support to managers at all levels. Such an approach ensures that every segment of the organization is well-prepared to respond effectively, fostering a culture where resilience is a shared priority. Furthermore, it is essential for top management to raise awareness and offer specific support for managers regardless of whether their operations involve healthcare for infected patients or managers responsible for other departments within the organisation.

In the context of managers’ leading HCWs during a pandemic, one strategy aligned with the system theory approach and integrated into a practical perspective on managerial work is the design of work roles that grant autonomy to HCWs, thereby enabling them to proactively manage their daily operative work situations [16]. That approach effectively enhances employee empowerment, when employees are empowered, they are more likely to take initiative and contribute innovative solutions, which reduces the need for oversight. This not only lightens the managerial workload but also fosters a more dynamic and resilient team environment [23]. Furthermore, instead of imposing rigid leadership models, there is a need to encourage the development of leadership approaches among managers, with a focus on tailoring those approaches to specific contexts, including pandemic crisis management or routine care management. Our study’s results indicate that many managers are comfortable with empowered decision making responsibilities. Managers additionally perceived that top management operated under the assumption that managers possessed the requisite competence to effectively manage and provide sustainable leadership within their respective departments and units. A previous study has shown that line managers exposed to strict top-down management control experienced reduced autonomy [29]. Therefore, when managers are trusted and granted greater autonomy, it ought to create additional room for manoeuvre, allowing them to adapt and respond more effectively to dynamic challenges.

In our study, we found parallels with the findings of Ozmen and Arslan Yurumezoglu [30], who identified a comparable experience among nurse managers, who faced challenges with the weight of decision-making, acknowledging potential impact on the safety of HCWs. Moreover, our findings endorsed the challenges identified in Ozmen and Arslan Yurumezoglu [30] study, were nurse managers found to bear high workload during the pandemic, encountering difficulties in efficiently coordinating hospital organisations and expressing concerns about shortcomings in allocating resource management. As pointed out in our previous publication on describing the managers situation being relieved of work tasks would have supported them in their strained work situations [4]. Further expressed by Ozmen and Arslan Yurumezoglu [30] managers faced excessive workloads, along with the fact that nurse managers shouldered numerous additional responsibilities that arose because of the pandemic. To fulfil these duties, they extend their working hours, and the scope of their responsibilities expanded considerably as they encountered various challenges along the pandemic’s unpredictable trajectory. Moreover, our study identified the daily challenges of managing operative daily work during crisis, emphasizing the need for adaptive strategies, and streamlined processes to maintain efficiency and effectiveness. Our findings reveal that top management integrated a new approach with centralised crisis management. However, based on these findings, top management ought to consider prioritizing more support for managerial work in a more structured manner, focusing on the themes identified in the results to address their needs effectively.

Even if our results show that both groups of managers invested a great deal of time in facilitating support and wellbeing among HCWs in their daily operative work, non-COVID- 19 managers dedicated even more focus to supporting their HCWs wellbeing. HCWs in the COVID- 19 departments had more knowledge about the pandemic and thus a more holistic view on the situation, whereas the HCWs in non-COVID departments did not have a full view on the situation. This finding suggests that HCWs in COVID- 19-focused departments may have had more capability to adapt and shape their work environments compared to those in non-COVID- 19 departments, highlighting the dynamics of manoeuvrability within organisation systems, underscored by Dellve and Eriksson [16].

One of the challenging responsibilities faced by the managers was effectively managing the information overload and determining its relevance for their HCWs and departments. As Dellve and Eriksson [16] discovered, the managers would have benefited from more targeted information, along with important factors such as balanced information and communication, in order to foster active engagement among HCWs.

Conflicts between hospital departments within are common issue that can arise due to differences in opinions on the best evidence of healthcare, misunderstandings, and stress during a pandemic. To manage conflicts, it is important to establish a code of conduct and uphold codes of ethics to keep patients and HCWs safe. In a related study encompassing all HCWs, three key themes emerged: problem-solving orientation, organisational learning, and information and communication [31]. These findings underscore the need to support managers across various departments, including ones providing pandemic healthcare and ordinary healthcare, in their role as catalysts for HCWs’ development and learning of new skills. Managers promote learning through collaboration, teamwork, idea sharing, and the improvement of practice to facilitate employees’ adaptation to developing healthcare practices. Managers experienced challenges in managing daily operative work, identifying areas for improvement. One support function, to facilitate daily operative work, was the IT department, which was proactive, innovative, and supportive in creating new ways of working. Insights into learning and development for future crises, ought to be clearer involvement from all support functions as a part of enhancing the dynamics in the organisational strategy. The managers expressed that the HR department ought to have expertise on how to handle HCWs during crises like pandemics, but it appears that everyone underestimated the HCWs’ fear of being infected or contagious, along with their worries [6]. The managers in both groups experienced that the HCWs were worried about the new situation that they faced, and the managers felt that a large amount of their time was spent listening to and supporting HCWs. These findings are supported by previous research indicating that managers had to devote much of their time to supporting HCWs during the COVID- 19 pandemic [5, 7, 32].

Both groups of managers reported gaining valuable insights into how to navigate future pandemics and enhance their preparedness for them. However, managers also expressed astonishment at the absence of a clear plan from top management for handling such crises. To effectively manage the work environment within hospitals during a long-term crisis, managers would have greatly benefited from having a strategic plan that balances the allocation of limited resources while minimising harm to individuals [33, 34], the for readiness. As confirmed by Gillberg et al. [31], HCWs’ displayed a problem-solving mindset and a desire to be proactive. Effective communication played a pivotal role in learning and development, and managers need to support innovations and make rapid decisions that lead to improvements. To that end, managers need to enhance trust-building efforts among HCWs and across organisational levels [16] and involve other stakeholders within the hospital such as patients and their relatives. Encouraging a culture of open communication and feedback is essential to identifying areas for improvement and sharing best practices. As found in a cross-sectional descriptive study conducted in Denmark during the early stages of the pandemic, managers with a formal management education and more than 5 years of work experience were more likely to possess the necessary managerial competences required for effective decision-making and collaboration in the context of managing the COVID- 19 pandemic.

The findings of our study’s deliver a clear message to the top management at hospitals, emphasising the prioritisation of the educational qualifications of current and future managers and underscoring the importance readiness and of providing additional support to managers during future pandemics. Research highlights how organisations should develop strategies and tools to prevent frontline managers from becoming caught in a difficult position between HCWs, patients, and strategic leaders [21]. By implementing those strategies, hospitals and their managers can start to foster important levels of competence among HCWs and adapt to the changing circumstances imposed by long-term crises. Those efforts, in turn, can improve patient safety, outcomes, and the overall quality of care.

Although the focus of the top-management and organisation was emergency care during pandemics, the long-lasting nature of such crises significantly impacts ordinary care and strains HCWs in ways that lead to immense suffering. Previous research has also highlighted the challenges hospital organisations faced when trying to balance the care needs of COVID- 19 patients and non-COVID- 19 patients [3]. Crises require immediate and effective action to minimise harm, protect lives and property, and restore normal operations as soon as possible [13]. However, major disasters and pandemics necessitate more complex, long-lasting, and methodical decision-making processes [33].

Hospital care was the focus of COVID- 19 treatment in Sweden, with the aim to have enough hospital beds and intensive care places for those people in need of hospital care. Unlike other countries, Sweden adopted a less restrictive strategy with more recommendations than prohibitions. The aim was to balance the reduction of transmission with the preservation of personal freedom and societal functions. However, this approach also resulted in high initial mortality, high pressure on hospitals, and inadequate preparedness, as well as increased workload, stress, and ethical dilemmas for HCWs [35]. A review of the Swedish COVID- 19 Commission’s reports revealed the strengths and weaknesses of the country’s distinctive approach and suggested several areas for improvement in pandemic preparedness at different levels of governance, including HCWs´ mental health, legal aspects, material resources, and organizational strategies [36]. Our findings underscored that to manage hospital capabilities, there should be a well-communicated plan with permission for the management to make top-down decisions and strict top-down management control to ensure that the hospitals´ capacities are used effectively during a crisis such as the pandemic. On the other hand, a recent editorial argued that nurses and other HCWs should have more influence on shaping the policies and interventions for COVID- 19 and future health crises, as they have valuable insights and experiences from working closely with patients [37]. This could have enhanced teamwork, interprofessional collaboration, and generated new insights into learning and improved development.

### Study strengths and limitations

In our study, we actively applied more of an iterative process while still adhering to all the prescribed steps outlined by Braun and Clarke [25] method of thematic analysis. Adhering rigidly to such methodologies we thought we might risk oversimplifying the rich, nuanced data, potentially concealing crucial contextual details and complexities essential for capturing the intended context. Criticism of highly structured processes like this suggests they could lead to mechanical analyses rather than reflective, interpretive, and iterative ones, potentially compromising the depth and authenticity of our findings [25]. We recognize the importance of demonstrating sufficient rigor in qualitative research to inspire confidence in our conclusions. Therefore, we utilized a rigorous, iterative coding process for thematic analysis, prioritizing methodological integrity over data saturation. This approach, which established trustworthiness through systematic theme refinement focusing on credibility, dependability, confirmability, and transferability, aimed to capture nuanced insights through a comprehensive analysis of the available data [28]. Additionally, we followed the Standards for Reporting on Qualitative Research [26] ensuring our research process was well-documented, methodologically sound, and transparent, which in our study consequently enhanced the credibility, replicability, and trustworthiness of our findings.

In our study, we stratified managers who were leaders in COVID- 19 wards at their hospitals after they responded to a survey questionnaire that inquired about their experience working with patients with COVID- 19. While most managers may not engage in direct patient care, managers play a critical role in supporting and facilitating the work of HCWs who do. The assumption was that managers answered the question based on their context and with reference to which patients were hospitalised in their departments.

The survey did not include variables that could capture the work experiences of the respondents as managers or their span of control, which could have been relevant for the research question. Previous studies have suggested that these factors may affect the decision-making processes, leadership styles, and organizational outcomes of managers in the public sector [38]. The inclusion of this data could have enabled a more comprehensive analysis of the sub-themes and themes, their interrelations and implications.

One limitation of our thematic analysis was the lack of prolonged engagement with participants [25], which could have been achieved by conducting multiple interviews or surveys. Given the circumstances and the significant pressure faced by the participants, we decided against further disrupting them. To translate our findings into practical strategies for enhancing managerial support, future research should focus on developing and piloting interventions. These interventions should preferably employ pre- and post-assessments to evaluate their longitudinal effects on managerial wellbeing, job satisfaction, and leadership effectiveness, particularly within the context of simulated pandemic crises.

## Conclusion

The results highlight that while managers appreciated the new ways of operating the hospital during the pandemic, their experiences also revealed areas in need of improvement. It is important to acknowledge the different prerequisites for managers leading hospital departments caring for patients with COVID- 19 versus those that do not, as these different contexts shaped their experiences of crises management. Hospital managers play a crucial role in navigating the complexities of centralised crisis management, ensuring daily operative work, providing managerial support to healthcare workers, and fostering a culture of continuous learning and development. The insights gathered from these themes highlights the importance of effective management and the dynamics of managerial support during times of pandemics, which directly impact patient safety. These results contribute to the ongoing improvement of healthcare organisations in addressing unforeseen challenges, such as pandemics and broader existential crises, by emphasizing the managerial perspective on crisis response.

### Clinical and organisational implications

To effectively address the multifaceted challenges of hospital crises, such as pandemics, we recommend the following key actions to support managers:

- The findings highlight the need for comprehensive and strategic, pre-existing crisis management plans, with managers playing an active role in their development and refinement.
- Acknowledge the need of continues, clear and targeted information flow to support managers during challenging times.
- Provide opportunities for ongoing training in interprofessional teamwork and collaboration, including crisis-driven scenarios.
- Foster continuous dialogue to address existential crises and promote managers and HCWs wellbeing at all levels within the hospital organisation.
- Best practices for rapid resource allocation and staff support during pandemic-like events are urgently needed.

We propose a framework for healthcare organisations to effectively organize, prepare, and practice for crises such as pandemics, *(Supplementary file *2*; Proposal for building capable hospital organisations during a pandemic)*. The proposal for building capable hospital organisations aims to enable optimal resource utilisation for pandemic care and to maintain ordinary care during prolonged long-term crises. To safeguard the continuity of hospital healthcare during a pandemic, it is essential to develop an organizational plan for two parallel working tracks. Developing hospital organizations capable of managing future crises requires the simultaneous advancement of two interacting parallel tracks: pandemic crisis management and ordinary care management. These tracks should efficiently manage both pandemic-oriented and routine healthcare, with the aim s of concurrently handling diverse patient diagnoses, enhancing productivity and healthcare quality, and ensuring a safe work.

## Supplementary material

Supplementary Material 1.

Supplementary Material 2.

## Data Availability

The data that support the findings of this study are available on request from the corresponding author. The data are not publicly available due to privacy or ethical restrictions.

## Abbreviations

HCWs: Healthcare workers
COVID-19 managers: Managers overseeing departments caring for patients infected with COVID-19
non COVID-19 managers: Managers overseeing departments not involved in caring for COVID-19 patients
