# Supplementary material for: Managers’ experiences in leading healthcare workers in hospital departments during a pandemic: a qualitative study
Source: BMC Health Serv Res. 2025 May 13;25:690. doi: 10.1186/s12913-025-12759-w (PMC12076825; doi:10.1186/s12913-025-12759-w)
Supplement: Supplementary file 1 — Supplementary Material 1. [file 12913_2025_12759_MOESM1_ESM.docx]

Survey question that has been used for the qualitative study: “Managers' Experiences in Leading Healthcare Workers in Hospital Departments During a Pandemic: A Qualitative Study”

Authors: *Linda Ahlstrom, Nanna Gillberg, Ewa Wikström, Helle Wijk, Ingibjörg H. Jonsdottir, Alessio Degl’Innocenti, Sara L. Fallman.*

| **English (translation)** | **Svenska (original)** |
| --- | --- |
| **Age**  **29 years or younger**  30 - 39 years  40 - 49 years  50 - 59 years  60 years or older | Ålder  29 år eller yngre  30 - 39 år  40 - 49 år  50 - 59 år  60 år eller äldre |
| **Gender**  **Female**  **Male**  **Another alternative** | Kön  Kvinna  Man  Annat alternativ |
| Did you work with COVID-19 patients during the spring of 2020, when the COVID-19 pandemic was at its peak?  Yes, daily  Yes, several times  Yes, occasionally  No | Arbetade du med covid-19 patienter under våren 2020 när covid-19 pandemin var som  mest aktuell?  Ja, dagligen  Ja, flera gånger  Ja, någon gång  Nej |
| **Are you a manager?** Yes No | Är du chef?  Ja  Nej |
| ***Below are some specific questions for managers.*** | ***Nedan följer några specifika chefsfrågor.*** |
| What organisational prerequisites were important to you in your role as a manager during the spring of 2020, when the COVID-19 pandemic was at its peak? (For example, clarity in decisions and procedures, division of responsibilities, resources, support, participation, and flexibility) | Vilka organisatoriska förutsättningar har varit viktiga för dig i ditt arbete som chef under våren 2020 när covid-19 pandemin var som mest aktuell?  (Exempelvis tydlighet i beslut och rutiner, ansvarsfördelning, resurser, stöd, delaktighet och handlingsutrymme) |
| What organisational prerequisites have you lacked in your role as a manager during the spring of 2020, when the COVID-19 pandemic was at its peak? (For example, clarity in decisions and procedures, division of responsibilities, resources, support, participation, and flexibility) | Vilka organisatoriska förutsättningar har du saknat i ditt arbete som chef under våren  2020 när covid-19 pandemin var som mest aktuell? (Exempelvis tydlighet i beslut och rutiner, ansvarsfördelning, resurser, stöd, delaktighet och handlingsutrymme) |
| What positive and negative experiences did you have during the spring of 2020, when the COVID-19 pandemic was at its peak? | Vilka positiva respektive negativa erfarenheter har du upplevt under våren 2020 när covid-19 pandemin var som mest aktuell? |
