# Supplementary material for: Managers’ experiences in leading healthcare workers in hospital departments during a pandemic: a qualitative study
Source: BMC Health Serv Res. 2025 May 13;25:690. doi: 10.1186/s12913-025-12759-w (PMC12076825; doi:10.1186/s12913-025-12759-w)
Supplement: Supplementary file 2 — Supplementary Material 2. [file 12913_2025_12759_MOESM2_ESM.docx]

**Supplementary material File 2: Proposal for building capable hospital organisations during a pandemic.**

Moving forward, the findings in the research article “Managers' Experiences in Leading Healthcare Workers in Hospital Departments During a Pandemic: A Qualitative Study” suggest lessons learned to enhance hospital organisations during crises, all derived from managers’ experiences during the COVID-19 pandemic.

To safeguard the continuity of healthcare, quality healthcare, and the safety of both patients and Healthcare workers (HCWs), it is essential to develop an organisational plan for two parallel working tracks during a long-term crisis. Those tracks, referring to the organisation of healthcare processes or systems to efficiently manage pandemic-oriented healthcare and healthcare as usual, are designed to concurrently manage different types of patient diagnoses simultaneously, enhance productivity and the quality of healthcare, and ensure a safe work environment (Figure 1). The first track, focused on caring for patients affected by the pandemic, is characterised by managing the prioritisation of the needs of patients with the disease responsible for the pandemic, as well as the need for adequate Personal Protective Equipment (PPE) for HCWs, the implementation of effective measures of infection control, and the provision of specialised care for patients with the disease. The allocation of additional resources, including funding and staffing, may be necessary to support the first track. The second track, by contrast, focuses on ordinary care for patients with illnesses unrelated to the pandemic. In prioritising the needs of patients with other diagnoses, the second track includes the provision of ordinary care, including preventive care and follow-up care, and the management of chronic conditions. The continuity of healthcare for those patients is crucial to prevent the development of serious complications, to ensure optimal health outcomes, and to not make the wait list of patients even longer.

To ensure the success of delivering high-quality healthcare within the hospital organisation, it is essential to have effective communication and coordination between the different departments and teams within the hospital. That arrangement includes sharing information and resources, as well as the implementation of effective systems for patient transfer between departments, as means to use resources effectively. Ensuring adequate staffing levels and the allocation of resources is also critical to ensuring that both tracks cooperate effectively and efficiently. By prioritising the needs of patients with the pandemic-related disease while also ensuring the continuity of healthcare for patients without the illness, hospitals can ensure the safety of both patients and HCWs and provide high-quality healthcare during a long-term pandemic.

To comprehensively address such a crisis, one track should concentrate on crisis management, while the other track has to maintain business as usual. When confronted with situations such as the COVID-19 pandemic, the initial phase is characterised by its acute, short-term nature, during which all resources and attention are directed to addressing the pandemic. As both tracks progress through the crisis in parallel, optimal interactions and cooperation between pandemic-related care and ordinary care are essential. The presence of a well-defined strategic plan is also foundational for achieving successful crisis management. Beyond that, it is crucial to emphasise the need for tailored information for the two parallel tracks. Directives and information from the organisation should be finely tuned to the specific context to guarantee that the right amount of relevant information is effectively communicated to the appropriate recipients. Such customisation is essential to facilitate optimal interaction and coordination between the tracks of pandemic care and ordinary care.


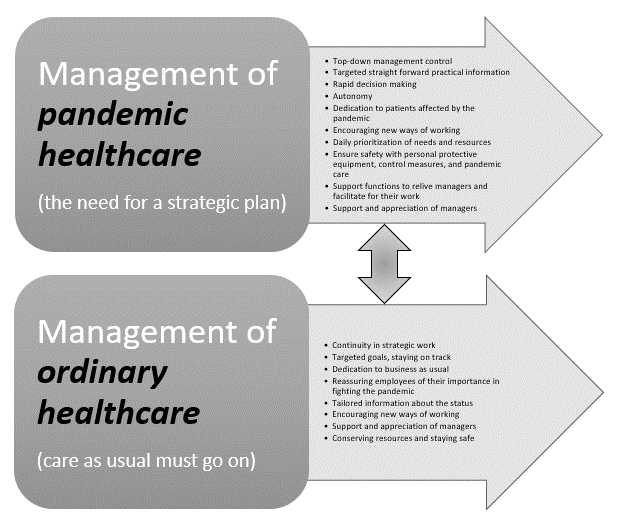


*Figure 1. Proposal for developing hospital organisations capable of leading two parallel tracks—management of pandemic healthcare and management of ordinary healthcare—in future pandemics.*

This article underscores the importance of enhancing hospital organisations’ preparedness and strategic considerations regarding operational strategies, along with other crucial aspects, to more effectively equip them for future crises and emerging threats. Managers emphasised the need to build capable hospital organisations and draw lessons from the COVID-19 pandemic to enhance emergency care for patients and minimise the impact on ordinary care. Despite a strong desire to avoid future pandemics, a significant global deficiency exists in the establishment of resilient healthcare systems (Rangachari et. al., 2020). In response, it is crucial to take immediate action and dedicate significant efforts to comprehending and managing pandemic-prone diseases and to preventing pandemics, and to those ends, global collaboration is needed (Haldane et al., 2021; Wang et al., 2020). Hopes aside, pandemics are an unavoidable reality that needs to be acknowledged. To validate our proposed approach, testing it within a proficient hospital organisation is essential. To that purpose, a feasible approach could involve simulating a pandemic scenario during hospital disaster drills.

This article provides a proposal based on lessons learned from the pandemic regarding the organisation of hospitals, policy formulation, and collaborations for building capable hospital organisations that are equipped to respond to pandemic challenges. Encouraging hospital organisations, their managers, researchers, and policymakers to engage in ongoing dialogue and knowledge sharing to drive continuous improvement in hospital organisations’ preparedness for pandemics is essential. The proposed approach with two tracks presented herein needs to be further investigated to be effective for future pandemic crises, and the results from the study could be used to encourage new research on the topic.

## References

Haldane, V., De Foo, C., Abdalla, S. M., Jung, A.-S., Tan, M., Wu, S., Chua, A., Verma, M., Shrestha, P., Singh, S., Perez, T., Tan, S. M., Bartos, M., Mabuchi, S., Bonk, M., McNab, C., Werner, G. K., Panjabi, R., Nordström, A., & Legido-Quigley, H. (2021). Health systems resilience in managing the COVID-19 pandemic: lessons from 28 countries. *Nature Medicine*, *27*(6), 964–980. <https://doi.org/10.1038/s41591-021-01381-y>

Rangachari, P., & Woods, J. L. (2020). Preserving organizational resilience, patient safety, and staff retention during covid-19 requires a holistic consideration of the psychological safety of healthcare workers. *International Journal of Environmental Research and Public Health*, *17*(12), 1–12. <https://doi.org/10.3390/ijerph17124267>

Wang, C., Horby, P. W., Hayden, F. G., & Gao, G. F. (2020). A novel coronavirus outbreak of global health concern. *The lancet*, *395*(10,223), 470-473. <https://doi-org.ezproxy.ub.gu.se/10.1016/S0140-6736(20)30185-9>
